# University of Louisville

**Published:** 1846-12

**Authors:** 


### THE WESTERN JOURNAL.

NEW SERIES.—VOL. VI.—NO. VI.

LOUISVILLE, DECEMBER 1, 1846.

With the present number is completed the fourteenth volume of the Western Journal of Medicine and Surgery, and at the conclusion of it we have but a few words to say to our readers. We have labored to render the Journal useful, and the marks of public favor afforded by the subscription list are such, that we are encouraged to persevere in our labors. The first number of the fifteeenth volume will appear punctually on the first day of the New Year. We have again to solicit from our professional brethren the contributions of their pens, and would particularly invite their attention to the unassuming department, in which we record "facts from correspondents." We shall be thankful for any medical facts communicated, no matter how brief, or how unstudied soever the form; and we trust that among our readers there are many who would be willing to take some pains to increase the number of our subscribers. With a trifling exertion on the part of those who take an interest in the Western Journal, we feel assured that the list of its subscribers might be doubled in the course of a single year. May we not hope to be witnesses of such a result, which, while it might be beneficial to the interests of our profession, would afford us fresh incentives to labor?

### UNIVERSITY OF LOUISVILLE.

It affords us great pleasure to say, that the first session of our University opens auspiciously. As our readers generally know, that which *was* the *Medical Institute of Louisville*, is now the Medical Department of the University of Louisville. The ultimate number of pupils in the last session of the former, was three hundred and forty five; the present number, constituting the first class of the University is three hundred and thirty-nine. A few more may be added, of such as have been kept back by sickness, or are but beginning the study of medicine. Thus the class is substantially the same as that of last year; but as the population of the interior of the continent is increasing, our class should also have increased. This we believe would have been the case, but for two or three causes which we shall proceed to assign.

1st. The session of 1844 had two hundred and eighty-six—that of 1845, three hundred and forty-five, an advance of fifty-nine.—Now this extraordinary increase was, in fact, that of two years. It was a rate of growth which, of course, could not be kept up.

2d. The volunteering for the Mexican war has, beyond a doubt, taken off students who might have been here.

3d. The general reduction in the amount of the cotton crop, is known to have kept away a number. These, we think, are the causes why the present is not larger than the preceding class.

That the class has risen a second time to more than three hundred, shows that the increase of last year was not owing to any temporary causes, as some persons at that time conjectured; and that it may be maintained at what it is, is rendered extremely probable. We can see no reason, indeed, why the number should not rise to three hundred and fifty, and continue at that average; for ten years are not sufficient for the full development of a school, and the present is but the tenth session of ours. When it was begun in 1837, the number of interior schools was six—it is now fourteen. Thus in ten years, seven new ones have been started, showing that ours has not attained its pre-eminence without a well contested and earnest competition.

We may be permitted again to mention, that the Trustees of the University of Louisville have erected a Law Department, of three professorships, and appointed Messrs. Pirtle, Duncan, and Loughborough, distinguished members of the Louisville bar, as professors. The first session of the school opened with the beginning of November. The number of pupils, we understand, is thirty-two, which may be regarded as decidedly promising for a first class. By the ordinances of the University they are entitled to gratuitous admission to all the lectures on Medical Jurisprudence delivered in the medical department.

